# Supplementary material for: Endangered wild salmon infected by newly discovered viruses
Source: eLife. 2019 Sep 3;8:e47615. doi: 10.7554/eLife.47615 (PMC6721791; doi:10.7554/eLife.47615)
Supplement: Supplementary file 1. [file elife-47615-supp1.docx]

| **Target** | **Forward Primer Sequence (5'-3')** | **Reverse Primer Sequence (5'-3')** | **Probe Sequence (5'-3')** |
| --- | --- | --- | --- |
| Salmon pescarenavirus 1 (SPAV-1) | CCTGCCTCTTTGCTCATTGTG | AGAAAAAGCTGTGGTACTTTAGAAAGC | ATCCGCCTAACGGTTGG |
| Salmon pescarenavirus 2 (SPAV-2) | AACATGAAGGGCGATTCGTT | CAGCCCGCGGACTGAGT | CAAGTGATGTAAGCTTG |
| Chinook Aquareovirus (CAV) | AACTTTCGGCTTTCTGCTATGC | GAGGACAAGGGTCTCCATCTGA | TTAATTGCGGTACTGCTC |
| Pacific salmon nidovirus (PsNV) | GGATAATCCCAACCGAAAAGTTT | GCATGAAATGTTGTCTCGGTTTAA | CGATCCCGATTATC |

Supplementary file 1

Primers and Taqman assays used in this study.
